# Germline pathogenic variants in *PALB2* and other cancer-predisposing genes in families with hereditary diffuse gastric cancer without *CDH1* mutation: a whole-exome sequencing study

**DOI:** 10.1016/S2468-1253(18)30079-7

**Published:** 2018-04-27

**Authors:** Eleanor Fewings, Alexey Larionov, James Redman, Mae A Goldgraben, James Scarth, Susan Richardson, Carole Brewer, Rosemarie Davidson, Ian Ellis, D Gareth Evans, Dorothy Halliday, Louise Izatt, Peter Marks, Vivienne McConnell, Louis Verbist, Rebecca Mayes, Graeme R Clark, James Hadfield, Suet-Feung Chin, Manuel R Teixeira, Olivier T Giger, Richard Hardwick, Massimiliano di Pietro, Maria O'Donovan, Paul Pharoah, Carlos Caldas, Rebecca C Fitzgerald, Marc Tischkowitz

**Affiliations:** a Academic Laboratory of Medical Genetics, University of Cambridge, Cambridge, UK; b Familial Gastric Cancer Study, Department of Oncology, University of Cambridge, Cambridge, UK; c Centre for Cancer Genetic Epidemiology, Strangeway's Research Laboratory, University of Cambridge, Cambridge, UK; d Cancer Research UK Cambridge Institute, University of Cambridge, Cambridge, UK; e Medical Research Council (MRC) Cancer Unit, Hutchison/MRC Research Centre, University of Cambridge, Cambridge, UK; f National Institute for Health Research Cambridge Biomedical Research Centre, Cambridge, UK; g Department of Histopathology, Cambridge University Hospitals NHS Foundation Trust, Cambridge, UK; h Department of Oesophago-Gastric Surgery, Cambridge University Hospitals NHS Foundation Trust, Cambridge, UK; i Precision Medicine and Genomics, Innovative Medicines and Early Development Biotech Unit, AstraZeneca, Cambridge, UK; j Peninsula Clinical Genetics Service, Exeter, UK; k West of Scotland Genetics Services, Glasgow, UK; l Cheshire and Merseyside Regional Genetic Service, Liverpool, UK; m Manchester Centre for Genomic Medicine, Manchester, UK; n Oxford Centre for Genomic Medicine, Oxford University Hospitals NHS Foundation Trust, Oxford, UK; o Clinical Genetics Service, Guy's and St Thomas' NHS Foundation Trust, London, UK; p West Midlands Regional Genetics Service, Birmingham, UK; q Northern Ireland Regional Genetics Centre, Belfast City Hospital, Belfast, UK; r Department of Gastroenterology, ZNA Jan Palfijn, Antwerp, Belgium; s Department of Genetics, Portuguese Oncology Institute of Porto, Porto, Portugal; t Institute of Biomedical Sciences, University of Porto, Porto, Portugal

## Abstract

**Background:**

Germline pathogenic variants in the E-cadherin gene (*CDH1*) are strongly associated with the development of hereditary diffuse gastric cancer. There is a paucity of data to guide risk assessment and management of families with hereditary diffuse gastric cancer that do not carry a *CDH1* pathogenic variant, making it difficult to make informed decisions about surveillance and risk-reducing surgery. We aimed to identify new candidate genes associated with predisposition to hereditary diffuse gastric cancer in affected families without pathogenic *CDH1* variants.

**Methods:**

We did whole-exome sequencing on DNA extracted from the blood of 39 individuals (28 individuals diagnosed with hereditary diffuse gastric cancer and 11 unaffected first-degree relatives) in 22 families without pathogenic *CDH1* variants. Genes with loss-of-function variants were prioritised using gene-interaction analysis to identify clusters of genes that could be involved in predisposition to hereditary diffuse gastric cancer.

**Findings:**

Protein-affecting germline variants were identified in probands from six families with hereditary diffuse gastric cancer; variants were found in genes known to predispose to cancer and in lesser-studied DNA repair genes. A frameshift deletion in *PALB2* was found in one member of a family with a history of gastric and breast cancer. Two different *MSH2* variants were identified in two unrelated affected individuals, including one frameshift insertion and one previously described start-codon loss. One family had a unique combination of variants in the DNA repair genes *ATR* and *NBN*. Two variants in the DNA repair gene *RECQL5* were identified in two unrelated families: one missense variant and a splice-acceptor variant.

**Interpretation:**

The results of this study suggest a role for the known cancer predisposition gene *PALB2* in families with hereditary diffuse gastric cancer and no detected pathogenic *CDH1* variants. We also identified new candidate genes associated with disease risk in these families.

**Funding:**

UK Medical Research Council (Sackler programme), European Research Council under the European Union's Seventh Framework Programme (2007–13), National Institute for Health Research Cambridge Biomedical Research Centre, Experimental Cancer Medicine Centres, and Cancer Research UK.

## Introduction

Gastric cancer is the fourth most common cancer globally. The best characterised inherited gastric cancer is the diffuse type, which has the hallmark of multiple foci of signet-ring cells.^1^ The term hereditary diffuse gastric cancer is used to describe families with a history of diffuse gastric cancer that meet the criteria of at least two cases of gastric cancer in first-degree or second-degree relatives regardless of age of onset (with one confirmed case of diffuse gastric cancer); one case of diffuse gastric cancer diagnosed before age 40 years; or a personal or family history of diffuse gastric cancer and lobular breast cancer, including one case diagnosed before age 50 years.^2^, ^3^

Germline mutations in the E-cadherin gene (*CDH1*) explain 25–30% of hereditary diffuse gastric cancer cases, with more than 100 pathogenic germline variants currently described within this gene.^4^ For families with hereditary diffuse gastric cancer and known pathogenic *CDH1* mutations, guidelines exist for risk assessment, disease management, surveillance (including regular endoscopies), and risk-reducing therapies (including prophylactic gastrectomy).^5^, ^6^ However, for families with no pathogenic variant in *CDH1*, the risk assessment is uncertain and, therefore, making decisions about and assessing the efficacy of risk-reducing strategies is challenging.

**Research in context**

**Evidence before this study**

Knowledge of factors causing predisposition to hereditary diffuse gastric cancer in families with no pathogenic variants in *CDH1* is limited by the rarity of the disease, which makes doing large-scale association studies difficult. In 2015, Hansford and colleagues described variants in DNA repair-related genes in 144 families with hereditary diffuse gastric cancer without *CDH1* pathogenic variants. These genes included *PALB2, BRCA2,* and *ATM*, which are associated with breast cancer risk. Further investigation of these genes, other known cancer-predisposing genes, and genes associated with DNA repair will aid in the disease management of families with hereditary diffuse gastric cancer without *CDH1* pathogenic variants, whose risk of disease development is currently unknown.

**Added value of this study**

This study is one of the largest germline, whole-exome sequencing analysis of families with hereditary diffuse gastric cancer without *CDH1* mutation to date. Both affected and unaffected individuals were recruited from families with hereditary diffuse gastric cancer, providing the opportunity to look for protein-affecting variants that segregate with phenotype. We used a unique approach to pathway analysis that involved clustering of physically interacting genes that were enriched for variants in these families and annotating them with a Gene Ontology term. Additionally, combining findings from this study with data from previously published studies allowed a more complete analysis of the role of the cancer predisposition genes *PALB2* and *BRCA2*.

**Implications of all the available evidence**

We identified a cluster of interacting genes involved in DNA repair that could be associated with predisposition to hereditary diffuse gastric cancer, in particular, *PALB2*. These findings should help guide future studies seeking to elucidate the clinical implications of genes that have not been previously associated with hereditary diffuse gastric cancer. Identification of these genes could provide families with hereditary diffuse gastric cancer without *CDH1* pathogenic variants with improved information about the risks associated with their disease and allow them to make informed decisions about risk reduction and disease management.

Other familial cancer syndromes that have been linked to gastric cancer predisposition include Lynch syndrome, which is characterised by mutations in DNA mismatch repair genes; Peutz-Jeghers syndrome caused by mutations in *STK11*; and Li-Fraumeni syndrome, which is associated with germline *TP53* mutations.^2^, ^7^, ^8^, ^9^ Diffuse gastric cancer does not appear to be over-represented in these syndromes, although this association has not been comprehensively studied.

Predicted pathogenic variants in the DNA double-strand break repair genes *ATM, BRCA2,* and *PALB2* have been identified in several families with hereditary diffuse gastric cancer.^4^, ^10^ However, given the rarity of these variants, the associated risk of diffuse gastric cancer is hard to quantify, and these variants are not used in routine clinical testing to aid management of these families.

We aimed to identify new candidate genes for predisposition to hereditary diffuse gastric cancer in families without pathogenic *CDH1* variants.

## Methods

### Study design and participants

In this whole-exome sequencing study, we recruited 28 individuals diagnosed with diffuse gastric cancer and 11 unaffected relatives from 22 families with hereditary diffuse gastric cancer that had tested negative for *CDH1* pathogenic germline mutations as part of the Familial Gastric Cancer study (MREC 97/5/32) and for whom blood and tumour samples were available. Families (including first-degree and second-degree relatives) were categorised as having hereditary diffuse gastric cancer on the basis of existing criteria.^2^, ^3^, ^6^

### Whole-exome sequencing and variant filtering

DNA was extracted from blood or saliva and prepared for 125-bp paired-end whole-exome sequencing using the Nextera Rapid Capture Exome Enrichment Kit (Illumina, San Diego, CA, USA). Sequencing was done on HiSeq-4000 or HiSeq-2500 platforms (Illumina, San Diego, CA, USA). Variant Call Format files were generated with a standard pipeline following Genome Analysis Toolkit (GATK) Best Practices recommendations for whole-exome data (appendix). The dataset was filtered to select uncommon (allele frequency <0·05 in the 1000 Genomes Project European sample) protein-affecting variants, including loss-of-function variants (stop site gained, stop site lost, start site lost, splice acceptor, splice donor, or frameshift), deleterious (predicted with Sorting Intolerant From Tolerant version 5.2.2) and damaging (predicted with Polymorphism Phenotyping version 2.2.2) missense variants, and inframe indels, that were observed in at least one of the 28 affected individuals. These filters were chosen to remove variants that were least likely to affect predisposition to hereditary diffuse gastric cancer. We considered the 11 unaffected family members separately on a per-family basis as a control group on which we did segregation analysis for identified candidate variants. We determined the allele frequency of all candidate variants in healthy controls (with no history of cancer) and allele counts in affected and unaffected individuals within families, and predicted downstream effects on the protein product.

Variants were aggregated into unique genes, which were then filtered to select those that contained at least one loss-of-function variant. We also removed the top 1% most variable genes, which were identified by the number of rare, protein-affecting variants per gene. These genes typically possess many rare variants within the healthy population and, therefore, are unlikely to have a role in predisposition to hereditary diffuse gastric cancer or other diseases. Variant-filtering and gene-filtering steps are summarised in the appendix.

To analyse copy number variants, we applied the XHMM algorithm to the gene set, using principle component analysis to normalise read depth across exomes and a hidden Markov model to identify regions with variation in read depth.^11^ Around a 50% decrease or increase in read depth was required for the variant to be considered for further analysis. Copy number variants were further explored in selected individuals with a CytoScan 750K genotyping array (Affymetrix, Santa Clara, CA, USA), according to the clinical protocol.

The results published here are in whole or part based on data generated by The Cancer Genome Atlas (TCGA), managed by the National Cancer Institute and the National Human Genome Research Institute. Controlled access data was requested and downloaded for the TCGA-STAD dataset, of which a subset of data from 88 cases of diffuse gastric cancer were analysed to further validate the role of the identified candidate genes in predisposition to diffuse gastric cancer.

### Gene-interaction network analysis

Gene-interaction network analysis was used to identify variant-enriched candidate genes with interacting protein products; non-antagonistic, physically interacting proteins might have a similar effect on cell function and, therefore, might produce a shared phenotype when mutated. The filtered genes were put through the GeneMANIA Cytoscape plugin version 3.4.1, which places physically interacting genes into clusters.^12^ A cluster was defined as a set of five or more physically interacting genes.

We used the PANTHER over-representation test (version 13.0) in the Gene Ontology Consortium enrichment analysis web-tool to assign Gene Ontology (GO) terms to clusters, applying the default Bonferroni correction for multiple testing.^13^ Of the significant terms highlighted in the analysis, the most significant term that encompassed between ten and 200 genes was selected, consistent with previous studies.^14^

Allelic counts of all filtered, loss-of-function variants within the selected GO terms (regardless of GeneMANIA Cytoscape clustering) were aggregated and contingency tables were drawn. Variants were also aggregated for each GO term over a comparably filtered set of genes from 503 European individuals in phase 3 of the 1000 Genomes study (appendix).^15^ We did a one-tailed Fisher's exact test using the R Stats package version 3.3.3 to test for enrichment of loss-of-function variants within each selected GO term in the families with hereditary diffuse gastric cancer compared with the 1000 Genomes European dataset. For this test, only one occurrence of a variant was counted per affected family. A link to the custom R scripts used for this analysis can be found in the appendix.

### Validation by Sanger sequencing

Candidate variants were validated by Sanger sequencing. Germline DNA from blood and extracted tumour DNA were quantified with the Qubit dsDNA HS Kit (Invitrogen, Carlsbad, CA, USA), and custom flanking primers were designed for each variant (primer sequences are shown in the appendix). DNA fragments were amplified by PCR and the products were sequenced on an ABI Genetic Analyser (Applied Biosystems Foster City, CA, USA) with BigDye Terminator version 3.1 (Invitrogen, Carlsbad, CA, USA), according to the manufacturer's instructions.

### Tumour immunohistochemistry and microsatellite instability analysis

We used the Ventana MMR IHC Panel (Roche, Indianapolis, IN, USA) to do immunohistochemistry analysis of known mismatch repair genes in available tumours from individuals in which variants in mismatch repair genes were identified. The panel includes antibodies against MLH1, PMS2, MSH2, and MSH6.

To analyse microsatellite instability, 5-μm formalin-fixed, paraffin-embedded tumour sections were mounted on glass slides for dewaxing and manual microdissection. DNA was extracted with the QIAamp DNA FFPE Tissue Kit (Qiagen, Hilden, Germany). We assessed the DNA for five standard microsatellite markers (BAT25, BAT26, NR21, NR24, and MONO27) using the Promega MSI Analysis System, version 1.2 (Promega, Madison, WI, USA). Poorly and moderately differentiated gastric tissue was compared with adjacent tumour-free tissue.

### Analysis of *PALB2* and *BRCA2* variants in published studies

We searched PubMed without language restrictions between Jan 1, 2015, and Dec 31, 2017, using the term “hereditary diffuse gastric cancer” to identify sequencing studies reporting loss-of-function variants in *PALB2* and *BRCA2* in hereditary diffuse gastric cancer probands with no detected pathogenic *CDH1* variants. We included only publications released after the initial report^4^ in 2015 of *PALB2* and *BRCA2* mutations associated with hereditary diffuse gastric cancer. For each of the four identified publications^4^, ^10^, ^16^, ^17^ and this study, we aggregated the allele counts of loss-of-function *PALB2* variants. The same counts were done across the 503 European samples from the 1000 Genomes Project and the 27 173 non-Finnish European individuals not in the TCGA from the Exome Aggregation Consortium (ExAC) control datasets.^18^ We removed the well characterised, non-pathogenic *BRCA2* polymorphic stop codon in c.9976A→T from all datasets. We did a one-tailed Fisher's exact test using the R Stats package version 3.3.3 to test for enrichment of loss-of-function *PALB2* or *BRCA2* variants in the families with hereditary diffuse gastric cancer compared with either control dataset.

### Role of the funding source

The funders had no role in study design, data collection, data analysis, data interpretation, or writing of the Article. EF, JR, and MT had access to the raw data. The corresponding author had full access to all of the data and the final responsibility for the decision to submit for publication.

## Results

A whole-exome sequencing dataset of 39 individuals from 22 families with hereditary diffuse gastric cancer without pathogenic *CDH1* variants (table 1) was filtered to select 3973 uncommon, protein-affecting variants that were aggregated into 2847 genes. Exclusion of the top 1% of highly variable genes, and retention of genes with at least one loss-of-function variant in affected individuals, resulted in a set of 732 genes (1228 variants). Eight highly variable genes were excluded, including *ANKRD36, CDC27, HLA-DRB1, HLA-DRB5, MUC3A, MUC4, MUC6*, and *OR4C5*. Additionally, the presence of affected and unaffected family members within our dataset allowed for selection of variants that segregated with phenotype on a per-family basis.

**Table 1:** Characteristics of 28 affected individuals in 22 families with hereditary diffuse gastric cancer without *CDH1* pathogenic variants

	Number of samples sequenced*		**Age of proband at diagnosis (years)**	**Cancers diagnosed in relatives of probands**†	**Candidate gene identified in proband**
	Affected	Unaffected			
1	2	0	41	Diffuse gastric cancer (44)‡, gastric cancer (57)	None
2	2	4	27	Peritoneal cancer, ovarian cancer (22), diffuse gastric cancer (24)‡, diffuse gastric cancer (28)	None
3	1	0	40	Gastric cancer (28), diffuse gastric cancer (48)	None
4	1	2	55	Breast cancer, lung cancer, laryngeal cancer, gastric cancer, and diffuse gastric cancer (44, 52)	*PALB2*
5	1	0	36§	Diffuse gastric cancer (37), lung cancer (54), colorectal cancer (57), breast cancer (50), diffuse gastric cancer (61), diffuse gastric cancer (79), lung cancer (83)	None
6	1	0	37	Breast cancer, gastric cancer (63), gastric cancer (64)	*RECQL5*
7	2	0	36	Colorectal cancer, breast cancer (43), diffuse gastric cancer (55)‡	None
8	1	0	47¶	Diffuse gastric cancer (44)	*MSH2*‖
9	1	0	44	Diffuse gastric cancer (28)	None
10	1	2	28	Breast cancer, gastric cancer (44), gastric cancer (47)	None
11	4	1	28	Signet-ring cells‡, Signet-ring cells‡, breast cancer (40s), diffuse gastric cancer (45)‡, prostate cancer (60s), colorectal cancer (75)	*ATR, NBN*
12	1	0	68	Lung cancer, gastric cancer (49), gastric cancer (50), gastric cancer (76)	*MSH2*‖
13	1	0	47	Gastric cancer, gastric cancer (50s), gastric cancer (60s)	None
14	1	0	23	Diffuse gastric cancer (40s), diffuse gastric cancer (46), thyroid cancer (30)	None
15	1	0	53	Gastric cancer (49), gastric cancer (67), gastric cancer (71)	None
16	1	0	37	Gastric cancer, breast cancer (54), breast cancer (65), colorectal cancer (66)	None
17	1	0	45	Diffuse gastric cancer (42)	None
18	1	0	48	Gastric cancer (44), gastric cancer (54)	None
19	1	1	35	Lung cancer, uterine cancer (65)	None
20	1	0	55	Gastric cancer (51), colorectal cancer (76)	None
21	1	1	28	Gastric cancer (53), breast cancer (76), gastric cancer (80)	*RECQL5*
22	1	0	30	Gastric cancer, diffuse gastric cancer (67)	None

In total, 39 individuals were sequenced, including 11 unaffected relatives. Numbers in parentheses indicate age in years at diagnosis.

* All probands were sequenced in this study.

† Includes both first-degree and second-degree relatives; age in years at diagnosis is in parentheses when known.

‡ Sequenced in this study.

§ This proband was also diganosed with colorectal cancer at age 47 years.

¶ This proband was also diagnosed with lobular breast cancer at age 36 years.

‖ No microsatellite instability was detected in tumour.

**Figure 1:**
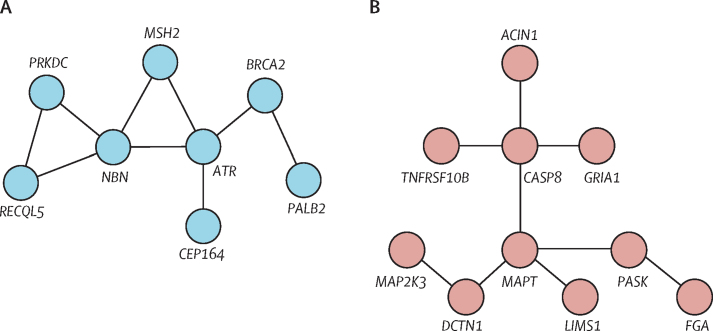
Gene clusters identified via gene interaction analysis Lines indicate a physical interaction, as assigned by the GeneMANIA plugin for Cytoscape.^12^ (A) Gene cluster to which the double-strand break repair GO term (GO:0006302) was assigned. (B) Gene cluster to which the negative regulation of extrinsic apoptotic signalling pathway via death domain receptors GO term (GO:1902042) was assigned. GO=Gene Ontology.

Gene-interaction network analysis of the 732 filtered genes identified two physical interaction clusters, to which GO terms were applied (figure 1). A cluster of eight genes was associated with the GO term double-strand break repair (GO:0006302; p<0·0001; figure 1A). A second cluster of ten genes was associated with the GO term negative regulation of extrinsic apoptotic signalling pathway via death domain receptors (GO:1902042; p=0·00517; figure 1B).

Loss-of-function variants within the filtered set of 1228 variants were aggregated under these two GO terms, including genes related to the GO terms that were not initially clustered by GeneMANIA. The double-strand break repair term was significantly enriched in families with hereditary diffuse gastric cancer compared with the 1000 Genomes European set (p=0·00051). By contrast, the apoptotic signalling pathway term was not enriched in families with hereditary diffuse gastric cancer (p=0·186), suggesting that the differences between the datasets in allele counts of DNA double-strand break repair genes cannot entirely be explained by technical differences that arise when using an externally produced control dataset.

Genes in the double-strand break repair GO term included *PALB2, MSH2, RECQL5, ATR,* and *NBN*, all of which were shown to be physically interacting in GeneMANIA (figure 1A). *BRCA2* was also a part of this set, but was disregarded from further study because it contained the well characterised, benign polymorphic stop codon c.9976A→T.^19^

**Figure 2:**
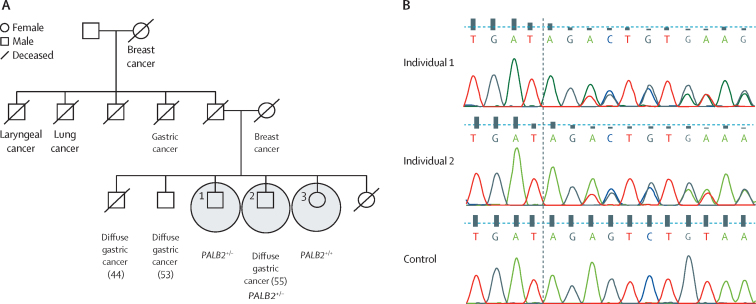
Pedigree and cancer history for family 4 (A) Whole-exome sequencing was done on the three circled individuals; age at diagnosis of cancer is shown in parentheses when known. (B) Chromatograms showing the *PALB2* frameshift variant (c.757-758TAG→T) in DNA from individuals 1 and 2 compared with control DNA.

Table 2 summarises the candidate variants. A heterozygous 2 bp frameshift deletion was identified in *PALB2* (c.757-758TAG→T [rs180177092, NM_024675.3]) in a patient from family 4 who was diagnosed with diffuse gastric cancer at age 55 years (figure 2). This loss-of-function variant at aminoacid position 253 is predicted to result in an early stop codon seven aminoacids downstream of the variant. Family 4 has a history of breast, lung, laryngeal, and diffuse gastric cancer (table 1, figure 2). Exome sequencing was also done on two unaffected siblings, one of whom also had the *PALB2* (c.757-758TAG→T [rs180177092, NM_024675.3]) variant. The affected proband had previously received treatment for *Helicobactor pylori* infection, but had tested negative at subsequent endoscopies.

**Table 2:** Candidate variants in six families with hereditary diffuse gastric cancer without *CDH1* pathogenic variants

	**Number of sequenced individuals**	**Gene**	**Variant**	**Consequence**	**Protein change**	**Minimum allele frequency**		**SIFT**	**Polymorphism Phenotyping**
						1000 Genomes European sample	ExAC non-TCGA European sample		
4	3	*PALB2*	c.757-758TAG→T	Frameshift deletion	Leu253fs	0	0	NA	NA
6	1	*RECQL5*	c.2806-2T→C	Splice-acceptor variant	NA	0	0	NA	NA
8	1	*MSH2*	c.967-968T→TCTCA	Frameshift insertion	Ser323fs	0	0	NA	NA
11	5	*ATR*	c.6075A→T	Stop site gain	Tyr2025X	0	0	NA	NA
11	5	*NBN*	c.1124+1G→C	Splice-donor variant	NA	0	0	NA	NA
12	1	*MSH2*	c.1A→C	Start site loss	Met1?*	0	0	Deleterious	Benign
21	2	*RECQL5*	c.2828C→T	Missense variant	Arg943His	0·002	0·014332	Deleterious	Probably damaging

SIFT=Sorting Intolerant From Tolerant. ExAC=Exome Aggregation Consortium. TCGA=The Cancer Genome Atlas. fs=frameshift. NA=not applicable.

* Human Genome Variation Society nomenclature to indicate loss of a start site without experimental evidence of a new start site.^20^

Two heterozygous loss-of-function variants were identified in the mismatch repair gene *MSH2*: a start site loss (c.1A→C [rs267607911, NM_000251.2]) in a patient from family 12 and a frameshift insertion of 4 bp (c.967-968T→TCTCA [NM_000251.2]) in a patient from family 8 (table 2; appendix). Both families had a strong history of gastric cancer; however, only DNA from the probands was available for sequencing, so segregation analysis could not be done. Heterozygosity of both variants was maintained in tumour DNA from the patients, as confirmed by Sanger sequencing. Tumours from both probands showed normal expression of MSH2 and other mismatch repair proteins by immunohistochemistry compared with adjacent tumour-free tissue (appendix), and neither tumour showed evidence of microsatellite instability (appendix). Both probands with *MSH2* variants had previously tested negative for *H pylori*.

**Figure 3:**
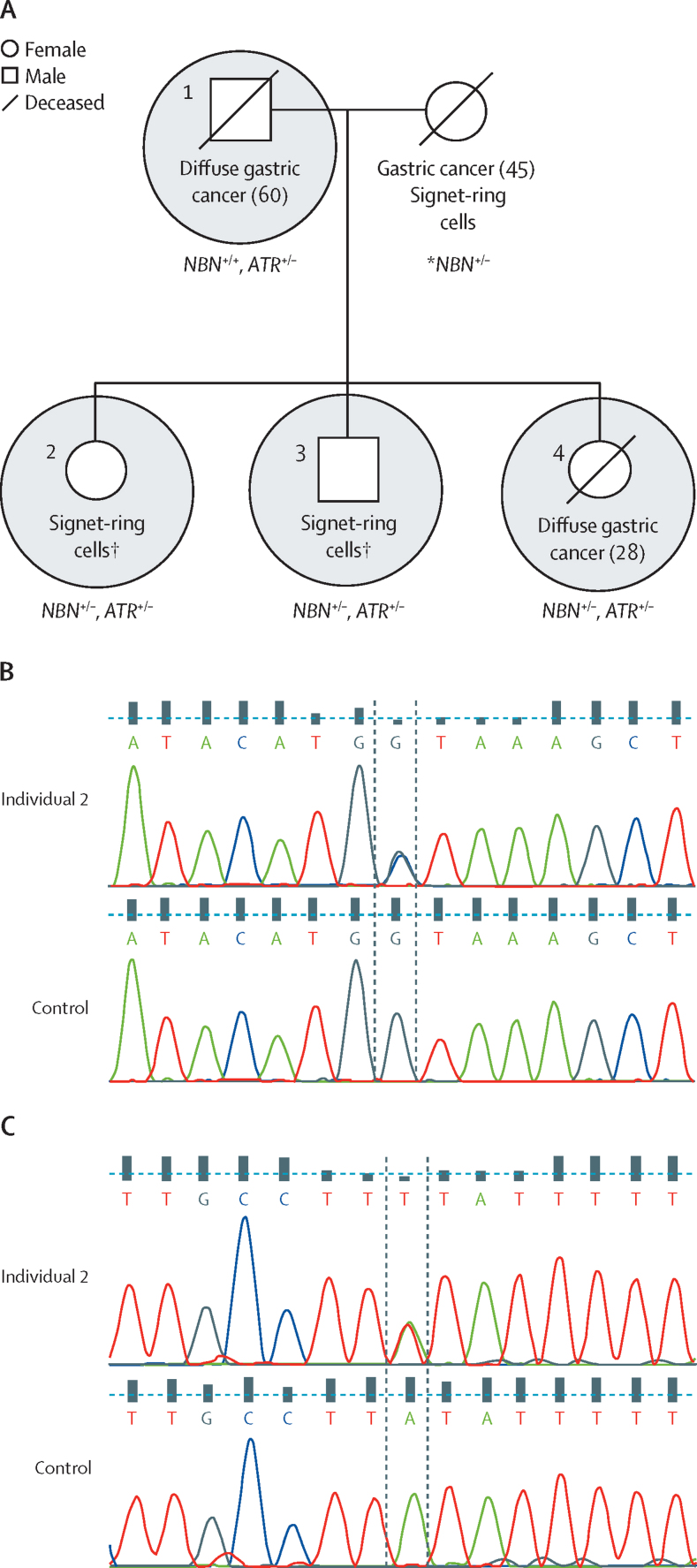
Pedigree and cancer history of family 11 (A) Whole-exome sequencing was done on the three circled individuals; the proband was patient 4. The presence of the variants in *NBN* (c.1124+1G→C) and *ATR* (c.6075A→T) among the four affected family members are shown. Age at diagnosis of cancer is shown in parentheses when known. *DNA was not available for the mother, and so the mother's genotype was assumed on the basis of the genotypes of the father and children. †These individuals underwent risk-reducing gastrectomies. (B) Chromatograms showing the *NBN* variants in DNA from individual 2 compared with control DNA. (C) Chromatograms showing the *ATR* variants in DNA from individual 2 compared with control DNA.

Heterozygous variants in the DNA repair genes *ATR* and *NBN*—a splice-donor variant (c.1124+1G→C [NM_002485.4]) in *NBN* and a predicted stop site-gain variant (c.6075A→T [NM_001184.3]) in *ATR*—were identified in the proband from family 11, who was diagnosed with diffuse gastric cancer at age 28 years (figure 3, table 2). Two siblings underwent risk-reducing gastrectomies, and subsequent pathological analysis of gastric tissue revealed the presence of signet-ring cells in both individuals. As such, these individuals were considered to be affected family members in this analysis. The father of the proband was diagnosed with diffuse gastric cancer at age 60 years and the mother had metastatic disease characterised by signet-ring cells, which suggests that she had primary gastric cancer. All individuals in this family whose DNA was sequenced tested negative for *H pylori*. The proband and both siblings were heterozygous for loss-of-function variants in both *ATR* and *NBN*. The splice-donor variant in *NBN* was not seen in the father, and so was presumed to have been inherited maternally (DNA was only available for the father). The *ATR* variant was identified in the father, in whom no clinically relevant copy number variants were found. An unaffected, second-degree relative in family 11 did not have either variant.

Two variants were identified in the helicase gene *RECQL5* in different families. One was a missense variant (c.2828C→T [rs200535477, NM_004259.6]) in the proband from family 21, who was diagnosed with diffuse gastric cancer at age 28 years, and the other was a loss-of–function, splice-acceptor variant (c.2806-2T→C [rs201841487, NM_004259.6]) in the proband from family 6, who was diagnosed with diffuse gastric cancer at age 37 years (table 2; appendix). Both of these families included individuals across three generations who were diagnosed with gastric cancer and breast cancer. The proband of family 6 tested negative for *H pylori*, and the *H pylori* status of the proband from family 21 was unknown. DNA from the father of the proband in family 21 was sequenced, and the missense variant in *RECQL5* was not found.

We did not explore variants in other genes within the double-strand break repair cluster because the variants did not segregate with the disease in families containing affected and unaffected members.

We analysed data from previous studies^4^, ^10^, ^16^, ^17^ to estimate enrichment of loss-of-function variants in *PALB2* and *BRCA2* in families with hereditary diffuse gastric cancer. A loss-of-function variant (c.1438A→T [rs1057520653, NM_024675.3]) was identified and reported to us by collaborators (Teixeira MR, unpublished), however this variant was not included in the analysis because it did not fit our search criteria. Five (2%) of the 329 probands tested in these studies (including the present study) had loss-of-function *PALB2* variants (table 3). By contrast, *PALB2* variants were identified in 26 (<1%) of 27 173 individuals in the non-TCGA, non-Finnish European ExAC database (p<0·0001) and in one (<1%) of 503 individuals in the 1000 Genomes Project European database (p=0·039). Loss-of-function *BRCA2* variants were not enriched in families with hereditary diffuse gastric cancer compared with ExAC (p=0·47) or 1000 Genomes Project (p=1·00) individuals. No loss-of-function *PALB2* variants were identified in a set of 88 cases with sporadic diffuse gastric cancer from the TCGA.

**Table 3:** *PALB2* variants identified in hereditary diffuse gastric cancer sequencing studies

	**Race**	**Patient ID**	**Diagnosis of proband (age at diagnosis in years)**	**Variant**	**Consequence**	**Protein change**
Hansford et al (2015)^4^	European	P124	Diffuse gastric cancer (45)	c.1193AC→A	fs deletion	Val398fs
Sahasrabudhe et al (2017)^10^	European	CG-12	Intestinal gastric cancer (69)	c.1240C→T	Stop-site gain	Arg414Ter
Sahasrabudhe et al (2017)^10^	European	CG-008	Diffuse gastric cancer (48)	c.1240C→T	Stop-site gain	Arg414Ter
Sahasrabudhe et al (2017)^10^	European	GM037589	Gastric cancer (46)	c.1240C→T	Stop-site gain	Arg414Ter
Sahasrabudhe et al (2017)^10^	European	CG-05	Diffuse gastric cancer (50)	c.3201+1G→T	Splice-site variant	NA
Sahasrabudhe et al (2017)^10^	European	CG-039	Diffuse gastric cancer (47)	c.1882_1890delAAGTCCTGC	In-frame deletion	Lys628_Cys630del
Sahasrabudhe et al (2017)^10^	Latin American	CG-028	Intestinal gastric cancer (81)	c.1882_1890delAAGTCCTGC	In-frame deletion	Lys628_Cys630del
Sahasrabudhe et al (2017)^10^	Latin American	3CG-103	Mixed (79)	c.2753C→A	Missense	Pro918Gln
Fewings et al (this study)	European	GST_172_301	Diffuse gastric cancer (55)	c.757_758TAG→T	fs deletion	Leu253fs
Teixeira (unpublished)	European	GM048157	Diffuse gastric cancer (56)	c.1438A→T	Stop-site gain	Lys480Ter

None of the identified *PALB2* variants appeared in the 1000 Genomes Project European samples or in the Exome Aggregation Consortium European datasets. fs=frameshift. NA=not applicable.

## Discussion

We found predicted pathogenic (protein-affecting) germline variants in known cancer-predisposing DNA repair genes (including *PALB2, MSH2, ATR, NBN*, and *RECQL5*) in six (27%) of 22 families with hereditary diffuse gastric cancer. This finding reflects the increasing number of cancer phenotypes found to be associated with existing cancer-predisposing genes as genomic analyses extend to rarer cancer subtypes. For example, mismatch repair genes were initially associated with increased risk of colorectal cancer, but were subsequently associated with risk of developing gastric and pancreatic cancers, among others.^21^, ^22^

Simply identifying predicted pathogenic variants in known cancer-predisposing genes does not imply causality. For example, pathogenic germline variants in *MSH2* were not accompanied by altered expression of DNA mismatch repair proteins in tumour tissue in our study. To investigate causality, larger studies with matched controls are required, which is not typically feasible for rare diseases. The generation of large control datasets, such as ExAC and 1000 Genomes, can be used to strengthen possible associations, as we have shown in the case of *PALB2*. When combining our data with those from previously published relevant studies,^4^, ^10^, ^16^, ^17^ including those in which no *PALB2* variants were found, we saw a significant over-representation of *PALB2* (but not *BRCA2*) pathogenic variants in families with hereditary diffuse gastric cancer compared with ExAC and 1000 Genomes controls; however, this finding was much less significant when comparing with the 503 Europeans in the 1000 Genomes Project set than when comparing with the 27 173 individuals from the non-Finnish, non-TCGA ExAC dataset.^15^

In several of the cases described by Sahasrabudhe and colleagues,^10^ tumour molecular profiling was done and showed that carriers of *PALB2* mutations had mutational signatures indicative of defects in homologous recombination. PALB2 has an important role in homologous recombination during double-stranded DNA break repair through recruitment of BRCA2 and RAD51 to DNA breaks. Mutations in this gene are associated with an increased risk of breast and pancreatic cancers.^23^, ^24^, ^25^ Even within families carrying *PALB2* mutations, cases of diffuse gastric cancer are likely to be rare and could be masked by a larger number of sporadic gastric adenocarcinomas, which means that associations with certain cancer subtypes might be missed in epidemiological studies of these families unless the pathology of all reported cancers is known. For example, a recent study^26^ revealed that a rare serous subtype of endometrial cancer is over-represented in carriers of *BRCA1* variants, identifying a novel cancer association with a gene that has been intensively studied for more than 20 years.

*ATR* and *NBN* are also involved in initiating the response to double-strand DNA breaks. The *NBN* gene product (NBS1) associates with MRE11 and RAD50 to form a complex involved in the activation of the ataxia proteins ATM and ATR, which have roles in the recruitment of damage repair proteins, cell cycle regulation, and apoptosis. We identified single loss-of-function variants in *NBN* or *ATR* in the parents of the proband in family 11, both of whom had, or were suspected to have, diffuse gastric cancer. These variants were co-inherited in all three siblings in the family. Expression of either one of these variants might predispose family members to diffuse gastric cancer, although no other incidences of gastric cancer, and only one instance of late-onset prostate cancer, were noted in an extensive maternal and paternal family history. Slavin and colleagues^17^ also identified a stop site-gain variant in *ATR* in an individual with intestinal-type adenocarcinoma and a strong family history of gastric cancer.

The unusual cancer pattern seen in family 11 (with all immediate family members [siblings and parents], but no extended family members, of the proband affected) might be attributed to multi-locus, inherited neoplasia alleles syndrome, in which inheritance of pathogenic mutations in multiple cancer-predisposing genes leads to an atypical or severe phenotype.^27^ The close functional relationship between *NBN* and *ATR* in double-stranded DNA break repair could indicate a potential combinatorial effect of variants in these genes, as potentially suggested by the young age of diagnoses or the presence of signet-ring cells in the siblings carrying these variants. By contrast, double heterozygosity of mutations in the DNA repair genes *BRCA1* and *BRCA2* in patients with breast cancer was found to be no more deleterious than a single heterozygous mutation.^28^ Nevertheless, such double heterozygosity might have implications for genetic counselling that should be considered.

Genetic variants in genes involved in the mismatch DNA repair pathway are also associated with Lynch syndrome. A variant similar to the start-site loss variant that we identified in *MSH2* (c.1A→G) has previously been shown to have only a mild effect on protein function;^29^ thus, this variant should not be treated as a typical loss-of-function variant. This attenuated effect on protein function could be due to the presence of an alternative start codon and a second non-mutated *MSH2* allele. Increased microsatellite instability, a measure of decreased MSH2 function, has been shown in patients and tumours with the c.1A→G start site-loss variant.^29^ However, in tumours from both cases analysed here, MSH2 expression (detected by immunohistochemistry staining) was normal and no microsatellite instability was found. Although it is most likely that tumorigenesis was not caused by mismatch repair deficiency, we cannot rule out the possibility of a novel, non-mismatch repairmediated mechanism of carcinogenesis driven by variants in *MSH2*.

The helicase RECQL5 is important for prevention of aberrant homologous recombination and accumulation of double-strand DNA breaks, and thus for preservation of genome stability.^30^ A missense *RECQL5* variant was identified in the proband from family 21, and was not found in the proband's unaffected father. A splice-acceptor variant in *RECQL5* was identified in an individual in family 6 who was diagnosed with diffuse gastric cancer at age 37 years. Both family 6 and family 21 had a history of breast and gastric cancer.

Previous studies have explored the role of known cancer predisposition genes in individuals with hereditary diffuse gastric cancer who do not have known *CDH1* mutations. Sahasrabudhe and colleagues^10^ identified germline variants in *PALB2, BRCA1,* and *RAD51C* in families with diffuse gastric cancer. Hansford and colleagues^4^ described variants in *ATM, BRCA2, MSR1,* and *STK11,* as well as a frameshift deletion in *PALB2*. This group has also uncovered a role for the *CDH1*-related adhesion gene *CTNNA1*. Although we did not find any variants of interest in *ATM, BRCA1, BRCA2, CTNNA1, MSR1, RAD51C,* or *STK11,* an exploration of *PALB2* variants in all families with hereditary diffuse gastric cancer sequenced in recent studies showed enrichment of loss-of-function variants in these families compared with control datasets. This finding makes a case for inclusion of *PALB2* in genetic testing for families with hereditary gastric cancer without *CDH1* mutations, and it is possible that individuals who carry *PALB2* mutations might benefit from platinum-based chemotherapy and treatment with PARP inhibitors.^31^ However, the evidence is not yet sufficient to recommend surveillance of diffuse gastric cancer in carriers of *PALB2* mutations because the absolute risk is likely to be low in the absence of a family history.

Sporadic stomach cancers have been analysed as part of the TCGA study,^32^ and an association was identified between truncating *PALB2* mutations and sporadic stomach adenocarcinoma. Of the individuals with sporadic stomach adenocarcinoma and *PALB2* mutations from the TCGA database, we selected 88 individuals with diffuse gastric cancer, as described by Bass and colleagues,^33^ among which we did not identify any truncating *PALB2* variants. However, the average age at diagnosis for this cohort was 66 years, so this finding is perhaps not unexpected given the younger age of onset usually observed in hereditary cancers.

The rarity of patients with hereditary diffuse gastric cancer without pathogenic *CDH1* variants makes the collection of large datasets challenging. We used gene-interaction network analysis to prioritise candidate gene variants that co-segregated with disease phenotype and were likely to be involved in predisposition to hereditary diffuse gastric cancer on the basis of knowledge of the biology of the disease. This approach did not overcome the problem of low statistical power due to a small sample size, which is often seen with rare cancer datasets, but it did allow for selection of the most plausible candidates from the available data.

We attempted an additional analysis of copy number variants within this dataset using the XHMM algorithm.^11^ Although this analysis did not suggest any plausible candidates, at present, copy number variant analysis of germline whole-exome sequencing data is not validated and, therefore, some causal copy number variants could have been missed.

In summary, we found that rare, protein-affecting variants in DNA damage repair genes were enriched in families with hereditary diffuse gastric cancer without pathogenic *CDH1* variants compared with control datasets. Further studies of these genes in similar families are required to increase knowledge of the genetic basis of hereditary diffuse gastric cancer so that better informed decisions about risk reduction and management in affected family members can be made. Lastly, for many families with hereditary diffuse gastric cancer without pathogenic *CDH1* variants, the underlying cause remains unexplained even after whole-exome sequencing, and although whole-genome sequencing might identify some additional candidates in regulatory elements or structural variants, it seems unlikely that high-impact genes other than *CDH1* will be implicated in hereditary diffuse gastric cancer. Therefore, focusing on moderate-impact or low-impact cancer genes, such as *PALB2*, might be the way forward for future studies of genes associated with predisposition to disease in these patients.
