# Supplementary material for: Germline pathogenic variants in PALB2 and other cancer-predisposing genes in families with hereditary diffuse gastric cancer without CDH1 mutation: a whole-exome sequencing study
Source: Lancet Gastroenterol Hepatol. 2018 Apr 27;3(7):489–98. doi: 10.1016/S2468-1253(18)30079-7 (PMC5992580; doi:10.1016/S2468-1253(18)30079-7)
Supplement: Supplementary appendix [file mmc1.pdf]

THE LANCET
Gastroenterology & Hepatology

Supplementary appendix

This appendix formed part of the original submission and has been peer reviewed. We post it as supplied by the authors.

Supplement to: Fewings E, Larionov A, Redman J, et al. Germline pathogenic variants in *PALB2* and other cancer-predisposing genes in families with hereditary diffuse gastric cancer without *CDH1* mutation: a whole-exome sequencing study. *Lancet Gastroenterol Hepatol* 2018; published online April 26. http://dx.doi.org/10.1016/S2468-1253(18)30079-7.

<u>Supplementary material: Whole exome sequencing study to detect germline pathogenic variants in *PALB2* and other cancer-predisposing genes in *CDH1*-negative diffuse gastric cancer families.</u>

<u>Supplementary Materials and Methods:</u>

Bioinformatics pipeline for VCF generation

Fastq files underwent demultiplexing and standard QC checks using FastQC prior to trimming of Illumina adaptors and low quality bases using Cutadapt (ver 1.8.1). The BWA-MEM algorithm (ver 0.7.12) was applied to align reads to GRCh37. BAM files from multiple lanes were merged, sorted and pre-processed (removal of PCR duplicates, base quality recalibration and local realignment around indels) using Samtools (ver 1.2), Picard (ver 2.6.0) and GATK (ver 3.6.0). Variant calling was performed across the set with GATK Haplotype Caller with 10bp padding around Nextera Exome Rapid Capture targets.

Optimised hard filters were applied, including a VQSR truth sensitivity of 99·5% for SNPs and 97% for INDELs, an average 10x depth (variant DP) per sample and a QUAL threshold of 200. The QUAL threshold corresponded to a TiTv ratio of 2 as calculated by Samtools VCF-Stats. Multi-allelic variants were flagged and excluded for the purpose of this analysis. Only genotypes with quality (GQ) >20 and individual depth (genotype DP) in sample < 500 were retained for further analysis. Ensembl VEP annotations were applied to select protein-affecting variants: loss of function (stop gained, stop lost, start lost, splice acceptor variant, splice donor variant, or frameshift variant), inframe indels and missense variants that were simultaneously called deleterious and probably damaging by SIFT and PolyPhen respectively. Common variants (AF > 0.05 in European 1000 genomes) were excluded. The non-common protein-affecting variants were aggregated per gene; these genes were used for interaction analyses and prioritised as described in main methods and in scripts below.

Scripts generated for all analysis downstream of VCF generation can be found at the following link (https://github.com/elliefewings/Fewings_HDGC_exome_2018). VCF data can be downloaded from the following repository (https://doi.org/10.17863/CAM.17181)

Validation by Sanger sequencing

Custom primers were designed for each variant and are summarised in supplementary table 1. Primers were designed to be between 18 and 26 bases in length with a melting temperature of around 60°C. The UCSC In-Silico PCR tool was used to check specificity of primer binding. Due to their proximity, both *RECQL5* variants (c.2806-2T>C and c.2828C>T) were covered by one pair of primers.

Gene interaction network analysis – Control data

The 1000 genomes project was used as a control set to test for an enrichment of loss of function variants under selected gene ontology terms in HDGC. Variants from European phase-3 1000 genomes data were filtered to select 28,833 uncommon (European AF <0·05 in 1000 genomes), protein affecting variants (loss of function, predicted deleterious and damaging missense and inframe indels). Variants were aggregated into 11,796 genes, which were filtered to select those with at least one loss of function variant and remove the top 1% most variable genes. Variability was measured by the number of rare, protein affecting variants each gene contains; 3,634 genes containing 4,601 loss of function variants were retained. Aggregated allele counts for each selected gene ontology term were generated using these loss of function variants for further analysis.

<u>Supplementary Results:</u>

VCF generation and quality metrics

Samples were sequenced across five whole exome sequencing libraries. Data quality of aligned, merged BAM files was checked using metrics generated by Qualimap and Picard (supplementary table 3). The mean percentage of targets covered at 20x across all samples was 80.23%. All identified candidate variants were manually checked in BAM files using IGV for region coverage and appropriate percentage of reads supporting the alternative variant call. Additionally all candidate variants were validated successfully by Sanger sequencing.

Supplementary Tables and Figures:

Gene	Variant	Forward Primer 5'-3'	Reverse Primer 5'-3'
PALB2	c.757-758TAG>T	GGAGAGAGACTGTGTCTTTGGCACTG	AGAGGTTGCTTCCAGGCTAAGACTC
RECQL5	c.2806-2T>C and c.2828C>T	CGTGTTAGCCAGGATGGTCTCG	CATGAGGAGGTGAGCGTTAGCC
MSH2	c.967-968T>TCTCA	GCGGGGCTTAGTGGCGTG	GACATCGCACCCAGCCCC
ATR	c.6075A>T	CCATTGATGTGGAACCTGTGGCTAC	GATTACTGGGATGAAGGGTAGTGGGG
NBN	c.1123+1C>G	CCCGTCATAGATGCCCGCAG	GCAGAGTGGAGGAGCTGGGAC
MSH2	c.1A>C	ACCTGGTGGCAACCTACCCTTG	ACCCCCTGGGTCTTGAACACC

Supplementary table 1: Primers used to validate candidate variants by Sanger sequencing.

GO Term	Counts for minor alleles in HDGC	Counts for minor alleles in Controls	Counts for major alleles in HDGC	Counts for major alleles in Controls	One-tailed Fishers Exact P value
Double strand break repair (GO:0006302)	13	118	31	888	0·0005
Negative regulation of extrinsic apoptotic signaling pathway via death domain receptors (GO:1902042)	4	50	40	956	0·186

Supplementary table 2: Aggregated allele counts of non-common protein-affecting variants within the HDGC set and a European 1000 genomes control set.

Sample	Percentage Mapped Reads	Mean Insert Size	Mean Mapping Quality	GC%	Mean Coverage on targets (X)	Percentage at 20X Coverage on targets
GPQ_045_202	99.96%	170.63	57.42	48.58%	35.25	72.16%
GPQ_045_203	99.97%	150.41	57.37	48.64%	25.25	56.65%
GPQ_047_301	99.89%	185.51	57.07	48.60%	76.08	85.63%
GPQ_047_302	99.89%	182.58	57.06	47.71%	62.05	81.03%
GPQ_047_303	99.76%	100.68	56.76	48.61%	40.37	72.55%
GPQ_047_304	99.93%	151.03	57.06	48.04%	56.07	80.67%
GPQ_047_305	99.94%	133.57	57.02	46.80%	51.75	75.47%
GPQ_047_308	99.67%	107.89	56.78	49.33%	29.81	63.63%
GPQ_048_401	99.98%	184.18	57.43	48.89%	50.76	84.36%
GST_172_301	99.97%	198.75	57.40	49.24%	47.21	83.18%
GST_172_302	99.96%	180.50	57.36	48.91%	65.33	89.33%
GST_172_303	99.97%	146.01	57.36	48.39%	33.32	68.88%
GST_230_304	99.98%	148.69	57.35	53.37%	43.62	70.30%
GST_256_301	99.97%	176.90	57.43	48.48%	64.44	88.54%
GST_257_201	99.97%	205.06	57.41	53.43%	71.48	87.47%
GST_257_202	99.98%	118.26	57.26	50.38%	32.14	64.91%
GST_275_201	99.96%	194.56	57.44	49.31%	69.97	91.53%
GST_296_201	99.96%	207.70	57.47	49.19%	51.99	85.58%
GST_345_301	99.96%	136.39	57.33	48.49%	59.64	84.49%
GST_345_302	99.97%	208.28	57.47	49.23%	96.14	94.73%
GST_345_303	99.97%	197.11	57.44	49.34%	87.28	93.73%
GST_349_202	99.95%	117.72	57.38	47.74%	101.16	87.64%
GST_349_252	99.92%	176.85	57.05	46.74%	77.56	84.28%
GST_349_301	99.91%	164.34	57.08	46.68%	66.37	81.09%
GST_349_302	99.96%	154.59	57.12	46.53%	45.50	73.69%
GST_349_303	99.94%	158.02	57.03	47.29%	51.10	76.71%
GST_358_301	99.97%	142.11	57.27	49.71%	54.06	83.55%
GST_368_301	99.96%	213.02	57.41	49.58%	141.41	97.46%
GST_440_403	99.97%	177.69	57.37	49.01%	76.64	91.39%
GST_441_301	99.95%	117.92	57.38	47.72%	96.99	87.57%
GST_444_301	99.96%	151.16	57.12	47.72%	48.18	77.44%
GST_446_301	99.93%	161.09	57.07	49.59%	21.04	45.64%
GST_455_301	99.96%	169.64	57.10	48.88%	43.94	76.84%
GST_459_301	99.92%	198.13	57.14	49.61%	96.14	90.30%
GST_459_302	99.93%	182.49	57.10	48.33%	79.52	87.00%
GST_460_201	99.94%	174.23	57.13	48.97%	53.45	81.35%
GST_463_301	99.91%	174.42	57.06	48.18%	54.02	81.95%
GST_463_402	99.95%	123.09	56.98	47.44%	28.85	58.78%
GST_464_301	99.94%	137.65	57.48	48.05%	129.07	91.63%

Supplementary table 3: Quality metrics generated from aligned and merged BAM files

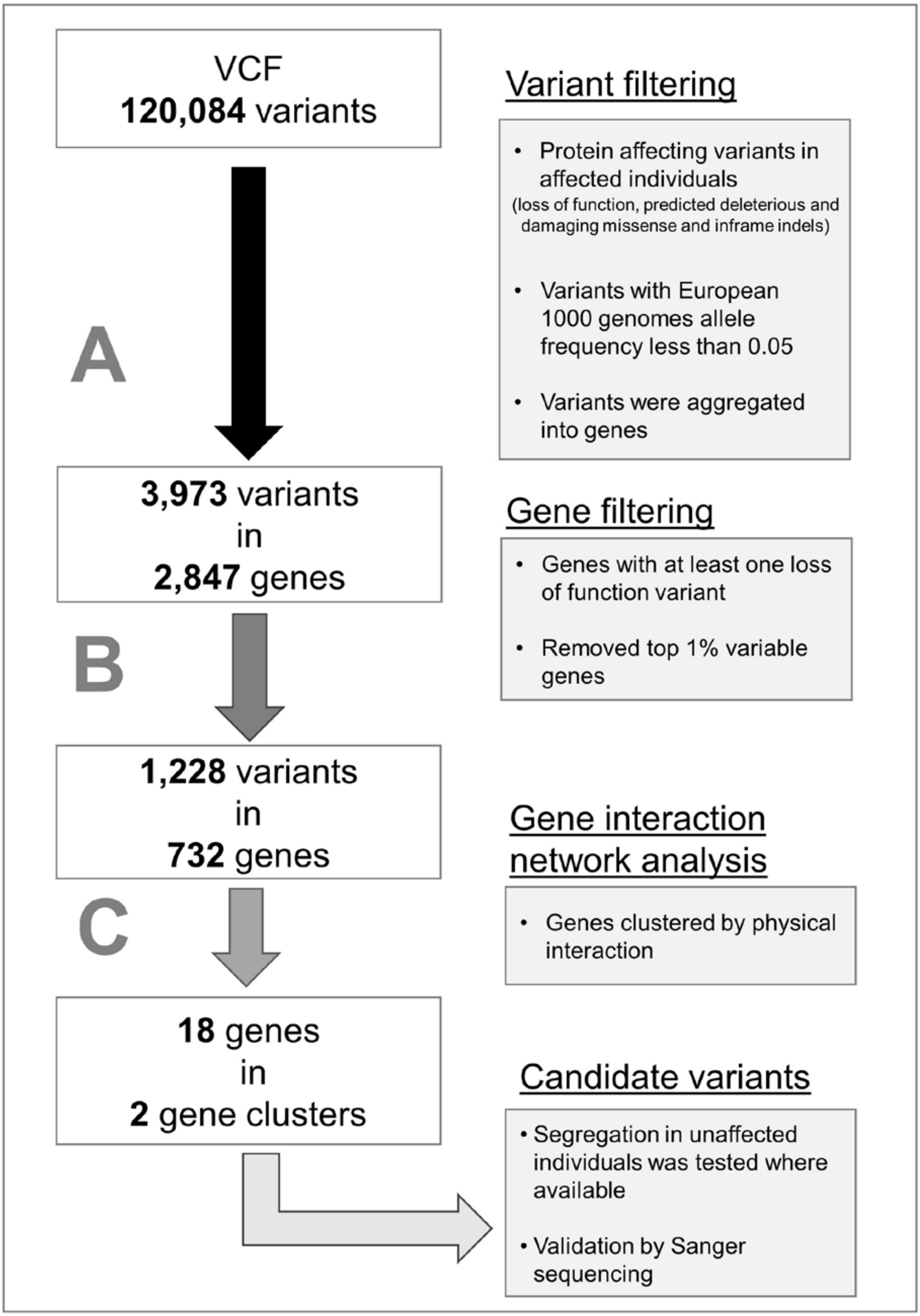

Supplementary figure 1: Variants filtering and analysis. A) variant filtering, B) gene filtering and C) gene clustering.

Gene	Variant	Consequence	Protein Change	Sample 1
MSH2	c.1A>C	Start loss	p.Met1?	A/C

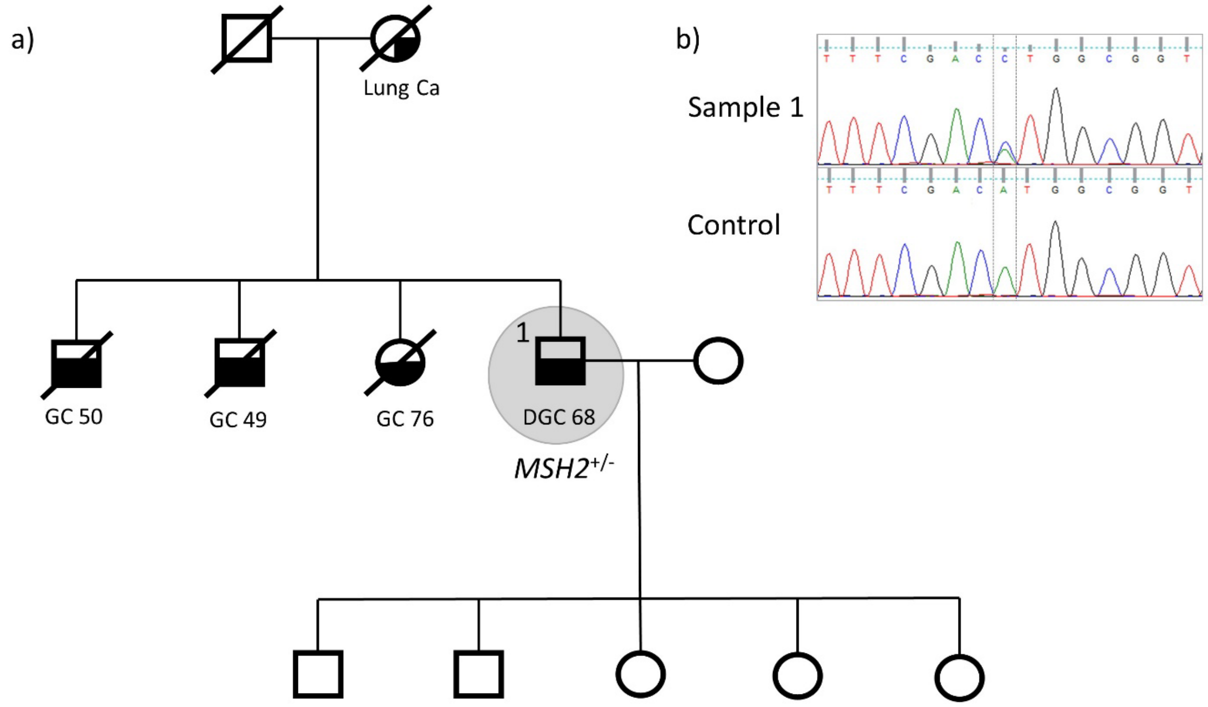

Supplementary Figure 2: a) The pedigree for family 12. b) Chromatograms showing the *MSH2* start loss variant in the proband against control DNA. Whole exome sequencing was performed on the circled sample, where shading indicates an affected individual.

Gene	Variant	Consequence	Protein Change	Sample 1
MSH2	c.967-968T>TCTCA	Frameshift variant	p.Ser323fs	T/TCTCA

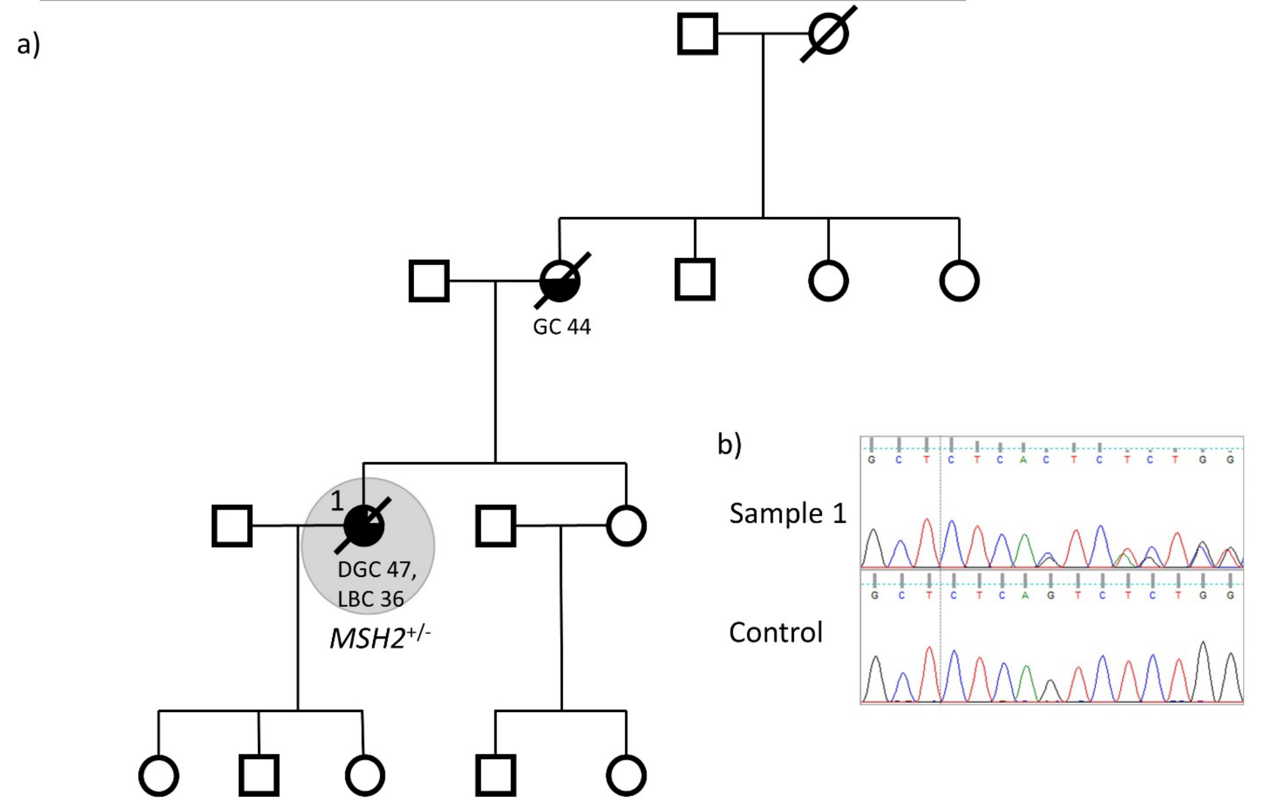

Supplementary Figure 3: a) The pedigree for family 8. b) Chromatograms showing the *MSH2* frameshift variant in the proband against control DNA. Whole exome sequencing was performed on the circled sample, where shading indicates an affected individual.

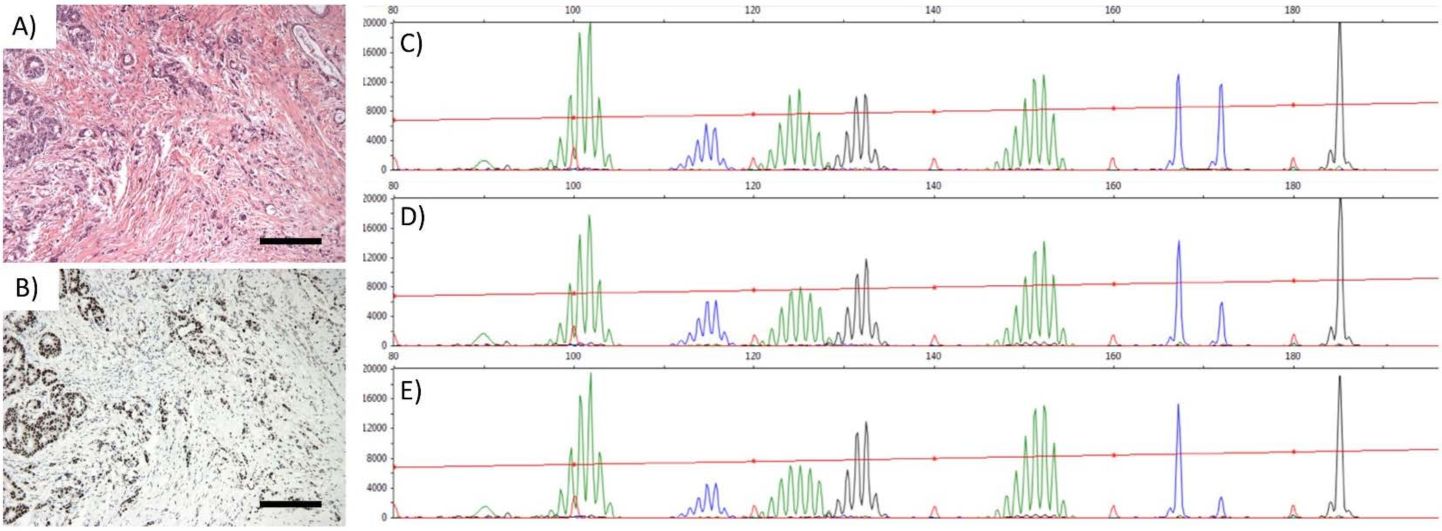

Supplementary Figure 4: Family 12 tumour analysis showing representative photomircographs (scale bar 0.2mm) showing a) hematoxylin and eosin b) normal MSH2 expression in tumours. Microsatellites are comparable across c) tumour-free adjacent tissue d) moderately differentiated gastric cancer tissue e) poorly differentiated gastric tissue.

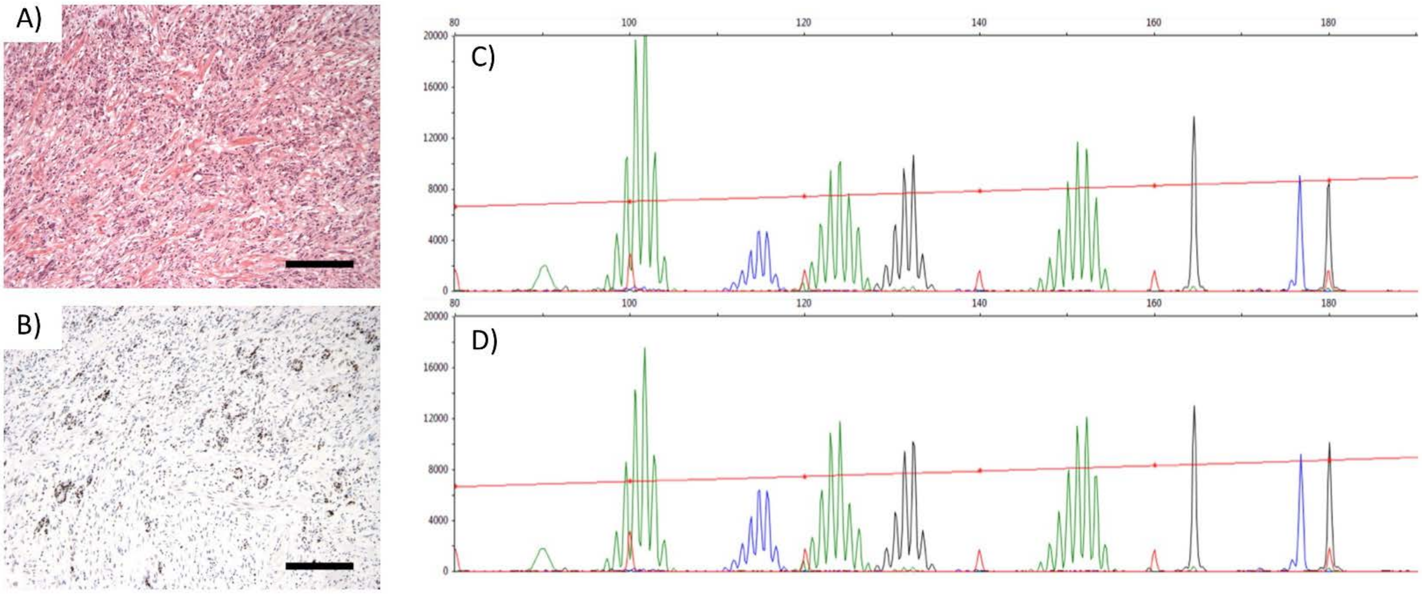

Supplementary Figure 5: Family 8 tumour analysis showing representative photomircographs (scale bar 0.2mm) showing a) hematoxylin and eosin b) normal MSH2 expression in tumours. Microsatellites are comparable across c) tumour-free adjacent tissue d) poorly differentiated gastric tissue.

Gene	Variant	Consequence	Protein Change	Sample 1	Sample 2
RECQL5	c.2828C>T	Missense variant	p.Arg943His	C/T	C/C

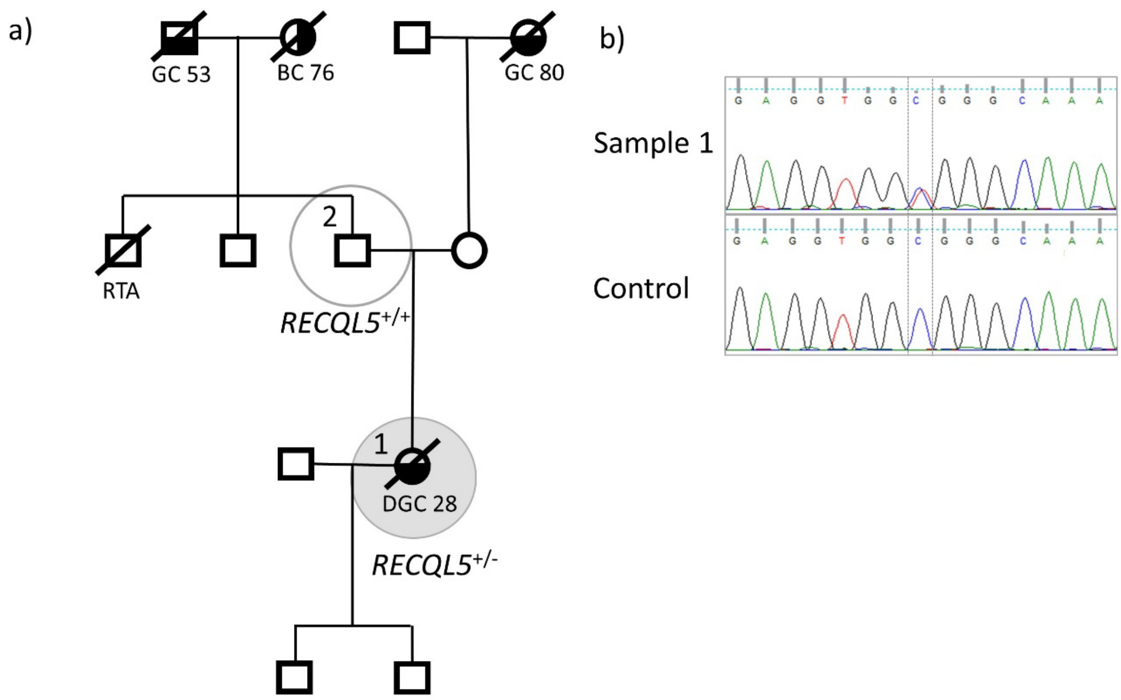

Supplementary Figure 6: a) The pedigree for family 21. b) Chromatograms showing the *RECQL5* missense variant in the proband against control DNA. Whole exome sequencing was performed on the circled sample, where shading indicates an affected and white indicates an unaffected individual.

Gene	Variant	Consequence	Protein Change	Sample 1
RECQL5	c.2806-2T>C	Splice acceptor variant		T/C

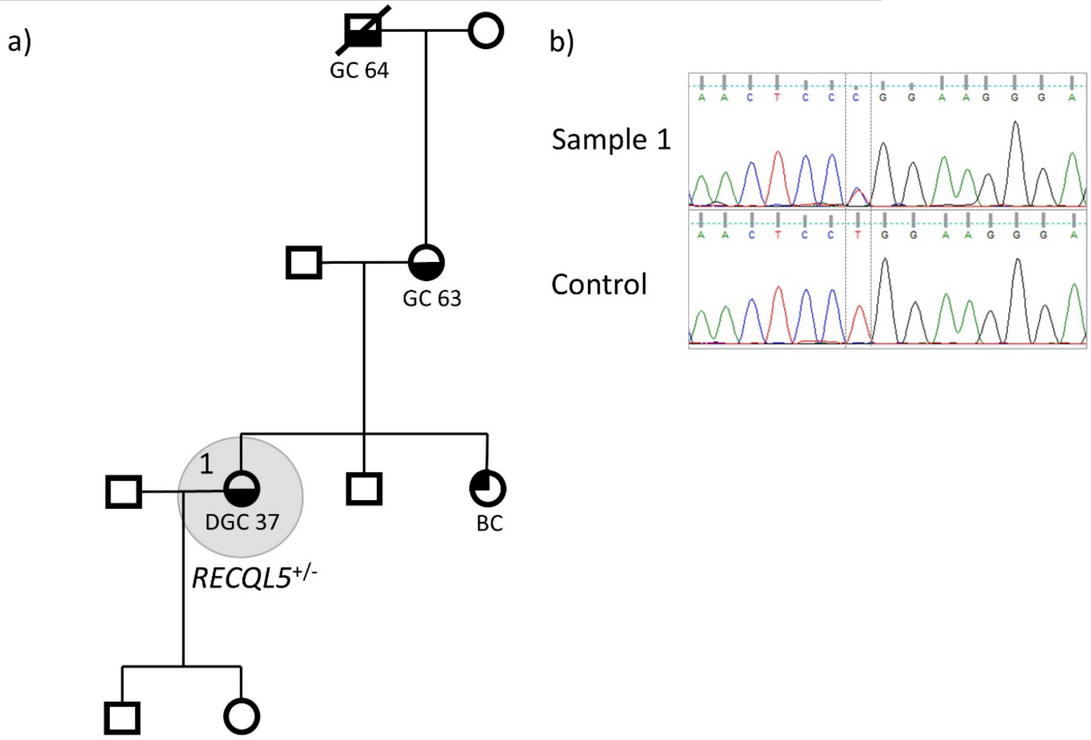

Supplementary Figure 7: a) The pedigree for family 6. b) Chromatograms showing the *RECQL5* splice acceptor variant in the proband against control DNA. Whole exome sequencing was performed on the circled sample, where shading indicates an affected individual.
